# Immediate angiographic control after intra-arterial nimodipine administration underestimates the vasodilatory effect

**DOI:** 10.1038/s41598-024-56807-7

**Published:** 2024-03-14

**Authors:** Charlotte Zaeske, David Zopfs, Kai Laukamp, Simon Lennartz, Jonathan Kottlors, Lukas Goertz, Henning Stetefeld, Marion Hof, Nuran Abdullayev, Christoph Kabbasch, Marc Schlamann, Michael Schönfeld

**Affiliations:** 1 Institute for Diagnostic and Interventional Radiology, University of Cologne, Faculty of Medicine and University Hospital Cologne, Kerpenerstr. 62, 50937 Cologne, Germany; 2 Department of Neurology, University of Cologne, Faculty of Medicine and University Hospital Cologne, Cologne, Germany; 3 Department of Neurosurgery, University of Cologne, Faculty of Medicine and University Hospital Cologne, Cologne, Germany

**Keywords:** Medical research, Cerebrovascular disorders, Neurovascular disorders

## Abstract

Intra-arterial nimodipine administration is a widely used rescue therapy for cerebral vasospasm. Although it is known that its effect sets in with delay, there is little evidence in current literature. Our aim was to prove that the maximal vasodilatory effect is underestimated in direct angiographic controls. We reviewed all cases of intra-arterial nimodipine treatment for subarachnoid hemorrhage-related cerebral vasospasm between January 2021 and December 2022. Inclusion criteria were availability of digital subtraction angiography runs before and after nimodipine administration and a delayed run for the most affected vessel at the end of the procedure to decide on further escalation of therapy. We evaluated nimodipine dose, timing of administration and vessel diameters. Delayed runs were performed in 32 cases (19 patients) with a mean delay of 37.6 (± 16.6) min after nimodipine administration and a mean total nimodipine dose of 4.7 (± 1.2) mg. Vessel dilation was more pronounced in delayed vs. immediate controls, with greater changes in spastic vessel segments (n = 31: 113.5 (± 78.5%) vs. 32.2% (± 27.9%), p < 0.0001) vs. non-spastic vessel segments (n = 32: 23.1% (± 13.5%) vs. 13.3% (± 10.7%), p < 0.0001). In conclusion intra-arterially administered nimodipine seems to exert a delayed vasodilatory effect, which should be considered before escalation of therapy.

## Introduction

Cerebral vasospasm continues to be a major contributor to morbidity and mortality in patients with aneurysmal subarachnoid hemorrhage^1^. Medical treatment with oral or intra-venous administration of the calcium-antagonist nimodipine together with blood pressure management aimed towards optimizing cerebral blood flow is usually the first step when vasospasm is expected; however, vasospasm can be refractory to these treatments^2^. Intra-arterial administration of nimodipine has been shown to be an effective treatment for cerebral vasospasm^3,4^ and can be performed as a salvage approach in the event of oral or intra-venous therapy failure^1^. Studies suggest that early and repeated intra-arterial treatment might be beneficial^5^. To date, the only option for treatment escalation is angioplasty with a balloon or a temporarily placed stent, which is usually limited to proximal vessels (> 2–3 mm in diameter) and which entails the risk of serious complications in approximately 5% of cases, including the risk of vessel perforation, which usually leads to severe morbidity or death^2,6–10^. Apart from this quandary, large-vessel vasospasm has been questioned as the main influence of patient outcome, as more recent hypotheses shifted the focus on mechanisms at a more complex combination of pathophysiological processes, including mechanisms such as early brain injury, microcirculatory disturbances, impaired autoregulation, and spreading depolarization^11^, which might be more accessible through medical treatment^12^.

Regarding the angiographic outcome, changes in vessel diameter and success of vasospasmolysis are often assessed immediately after completion of nimodipine infusion^10,13^. However, it is known that the effect of nimodipine sets in with some delay, even though there is little evidence for this in the current literature. We suspect that the maximum effect of nimodipine might be underestimated by an immediately performed angiographic control. Therefore, we evaluated the delayed angiographic effect of intra-arterial nimodipine as a salvage therapy for cerebral vasospasm after subarachnoid hemorrhage.

## Methods

### Patient inclusion

We retrospectively reviewed all cases of intra-arterial nimodipine treatment for subarachnoid hemorrhage-related cerebral vasospasm in a single institution in Germany during an inclusion period between January 2021 and December 2022. All procedures performed in this study were in accordance with the ethical standards of the institutional and national research committee and with the 1964 Helsinki declaration and its later amendments. Because of the retrospective design of the study, the local ethics committee (Ethics Committee of the University Hospital Cologne, Germany) waived explicit approval as well as the need for informed consent.

Inclusion criteria were a history of recent aneurysmal subarachnoid hemorrhage, the availability of digital subtraction angiography runs before and immediately after nimodipine administration as well as a subsequent delayed run to visualize the most severely compromised vessel at the end of the procedure, which is frequently performed in our institution to decide on further escalation of therapy. A total of 32 angiographies from 19 patients contained both immediate and delayed digital subtraction angiography runs and were thus selected for further analysis.

Baseline characteristics, such as demographic data (age, sex) as well as assumed cause of bleeding, day of ictus and Modified Ranking Scale (mRS) score at discharge were collected from patient charts and medical reports. Favorable outcome was defined as an mRS score from 0 to 2. Procedural data such as vessel selection and diameter, the time between the immediate and the delayed post-vasospasmolytic angiographic controls, and the amount and timing of nimodipine administration were taken from angiographic imaging data and angiographic reports. All authors had access to the raw data used to generate the database (patient charts and medical reports, angiographic imaging data, angiographic reports). Data cleaning was performed by checking for data duplications and data formatting. Correction of missing data or outliers was not necessary.

### Vasospasmolysis procedure

**Figure 1:**
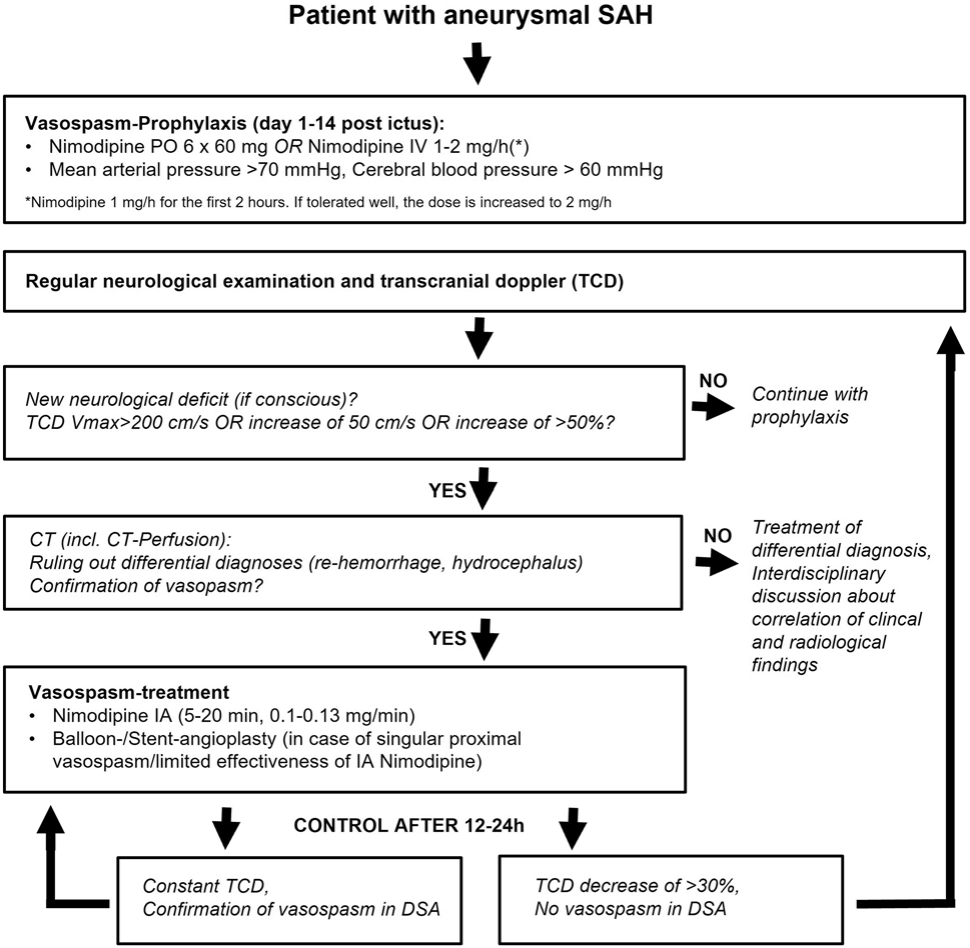
In-House standard operating procedure for diagnostic work-up, prophylaxis and therapy of cerebral vasospasm. *SAH* subarachnoid hemorrhage, *TCD* transcranial doppler, *Vmax* maximum velocity.

The diagnostic work-up, prophylaxis and therapy of cerebral vasospasm according to the in-house standard operating procedure are shown in Fig. 1. All procedures were performed under general anesthesia on a bi-planar angiography suite using a standardized approach. Nimodipine was infused using a perfusor device at a rate of 0.10–0.13 mg/min for 5–20 min into the cervical segments of the internal carotid artery or vertebral artery depending on the affected vessels under consideration of the demand for IV norepinephrine and additional oxygen to maintain intra-arterially measured blood pressure and oxygen saturation. Delayed digital subtraction angiography runs were performed at the discretion of the treating neurointerventionalist in cases of severe vasospasm as an alternative to immediate escalation of therapy, such as mechanical dilation. The delayed angiography runs were performed after treatment of other parts of the vasculature to obtain an estimate of potential cross-flow and to benefit from possible delayed effects of nimodipine. Therefore, no extubation or reintubation of the patient was performed during the procedure, nor was general anesthesia artificially prolonged to perform the delayed angiographies.

### Post-treatment angiographic evaluation

For each patient, the changes in vessel diameters were evaluated for one exemplary spastic and non spastic vessel segment. The spastic vessel segment was defined as the vessel segment which was mostly affected by vasospasm as per a qualitative determination on angiographic imaging data performed by a board-certified neuroradiologist (MS, 11 years of experience). The non-spastic vessel segment was defined as the first vessel segment proximal to the spasm that showed a normal caliber and absence of spasms. The vessel diameters were measured manually on angiograms in working projections. The percentage of dilation in relation to the baseline diameter was calculated for both immediate and delayed controls. The angiographic response was graded as insufficient if there was no improvement or only mild improvement in vessel caliber and there was a target for an escalation of endovascular therapy, or sufficient if all or most of the treated vessels improved and there was no target for an escalation of endovascular therapy.

### Statistical analysis

Results are presented as means including standard deviation. Since normal distribution of most of the data could not be confirmed by a Shapiro–Wilk test, further analysis was performed using non-parametric tests. For comparison of the relative vessel dilation in the immediate and delayed angiographic controls, the Wilcoxon matched pairs signed rank test was used. To analyze the effect of nimodipine administered after the immediate controls on progressive dilation between the immediate and delayed controls, non-parametric Spearman correlation was performed.

A two-sided p-value of < 0.05 was considered as statistically significant. Statistical analyses were performed using GraphPad Prism Version 9.3.0 (GraphPad Software Inc., San Diego, California, USA).

## Results

### Patient data

A total of 181 patients were treated for subarachnoid hemorrhage (including aneurysmal and nonaneurysmal subarachnoid hemorrhage) in our hospital during the inclusion period. Of these 181 patients, 63 received one or more angiographies for vasospasmolysis, resulting in a total of 262 angiographies. Data from 32 angiographies from 19 patients included a delayed angiography run and thus were chosen for further analysis, comprising nine female and ten male patients with a mean age of 53.7 (± 14.5) years. All included patients suffered from subarachnoid hemorrhage due to aneurysm rupture.

In total there were nine patients with one angiography, seven patients with two angiographies and three patients with three angiographies. Patients presented with a mean modified Rankin Scale score of 4.2 (± 1.8) at discharge, with a favorable outcome of.3 patients (15.79%). Further clinical details are presented in Table 1.

**Table 1:** Clinical information of the selected patients.

Age (years)	53.74 ± 14.46
Sex	
Female	10 (52.63%)
Male	9 (47.37%)
Site of aneurysm	
ICA	2 (10.53%)
MCA	5 (26.32%)
ACOM	6 (31.58%)
PCOM	1 (5.26%)
PCA	2 (10.53%)
PICA	3 (15.79%)
Hunt&Hess-grade	
1	4 (21.05%)
2	1 (5.26%)
3	3 (15.79%)
4	6 (31.58%)
5	5 (26.32%)
Fisher score	
1	1 (5.26%)
2	0 (0.00%)
3	12 (63.16%)
4	6 (31.58%)
Aneurysm treatment method	
Clipping	7 (36.84%)
Coiling	8 (42.11%)
Flowdiverter	2 (10.53%)
Intrasaccular flow disruptor (WEB-device)	2 (10.53%)
Number of performed angiographies for vasospasmolysis (total, during hospital stay)	5.62 ± 3.22
Modified Rankin Scale Score (at discharge)	4.15 ± 1.77

*ICA* internal carotid artery, *MCA* medial cerebral artery, *ACOM* anterior communicating artery, *PCOM* posterior communicating artery, *PCA* posterior cerebral artery, *PICA* posterior inferior cerebellar artery.

### Intervention

Interventional vasospasmolysis was performed a mean of 9.5 (± 3.7) days after ictus. During angiography, immediate controls were performed directly after termination of the nimodipine infusion, while the delayed controls were performed with a mean delay of 37.6 (± 16.6) min after nimodipine administration. In total, a mean dose of 4.7 (± 1.2) mg nimodipine was infused with an average amount of 1.8 (± 0.3) mg per vessel. Between the immediate and the delayed control, a mean of 2.5 mg (± 1.4) mg nimodipine was infused into vessels other than the one of the delayed control. In four cases, no nimodipine was administered between the immediate and the delayed control.

### Vessel dilation

**Figure 2:**
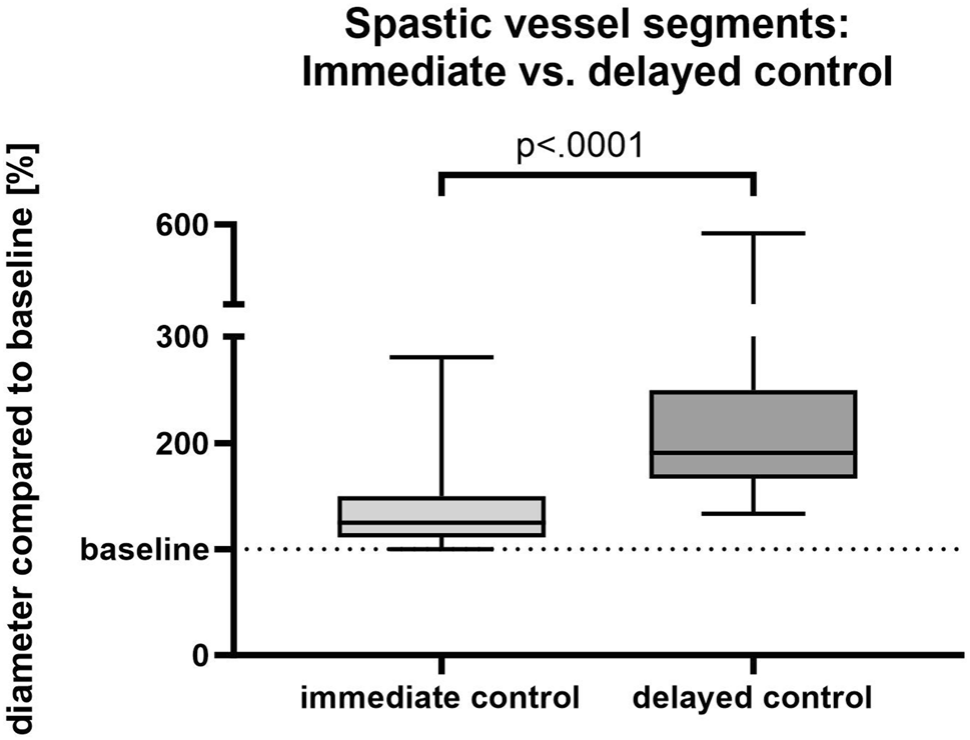
Changes in vessel diameter of spastic vessel segments.

Dilation of the spastic vessel segments was more pronounced in the delayed controls than in the immediate controls with an increase in diameter by 113.5% (± 78.5) vs. 32.2% (± 27.9) compared to the initial diameter (n = 31; p < 0.0001) (Fig. 2). One case had to be excluded from this analysis because the most spastic vessel segment was completely occluded both at baseline and in the immediate control but reopened in the delayed control 37 min after nimodipine infusion, prohibiting the calculation of the relative dilation of the vessel.

**Figure 3:**
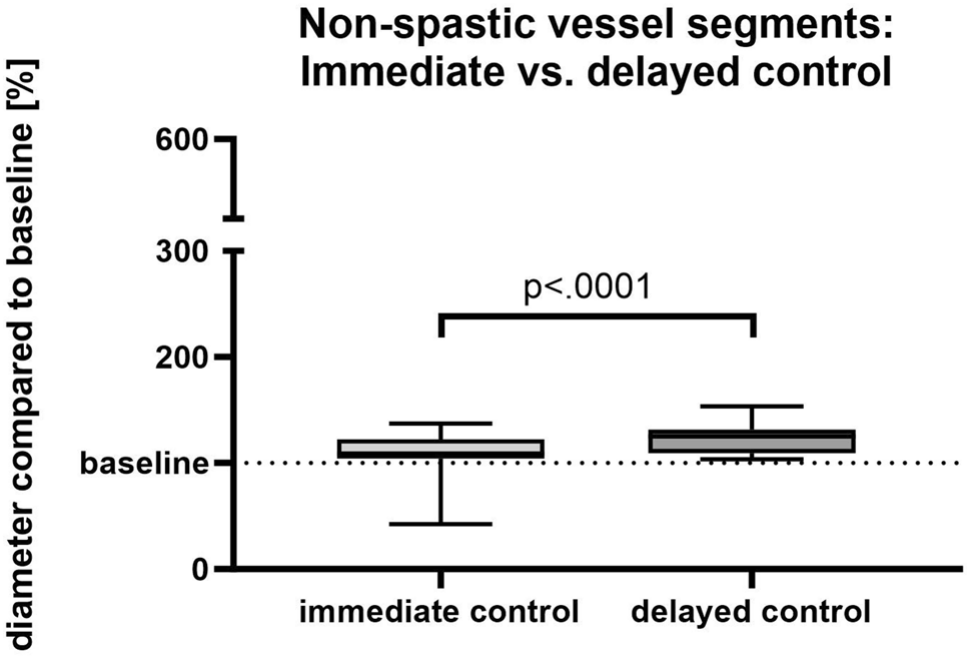
Changes in vessel diameter of non-spastic vessel segments.

**Figure 4:**
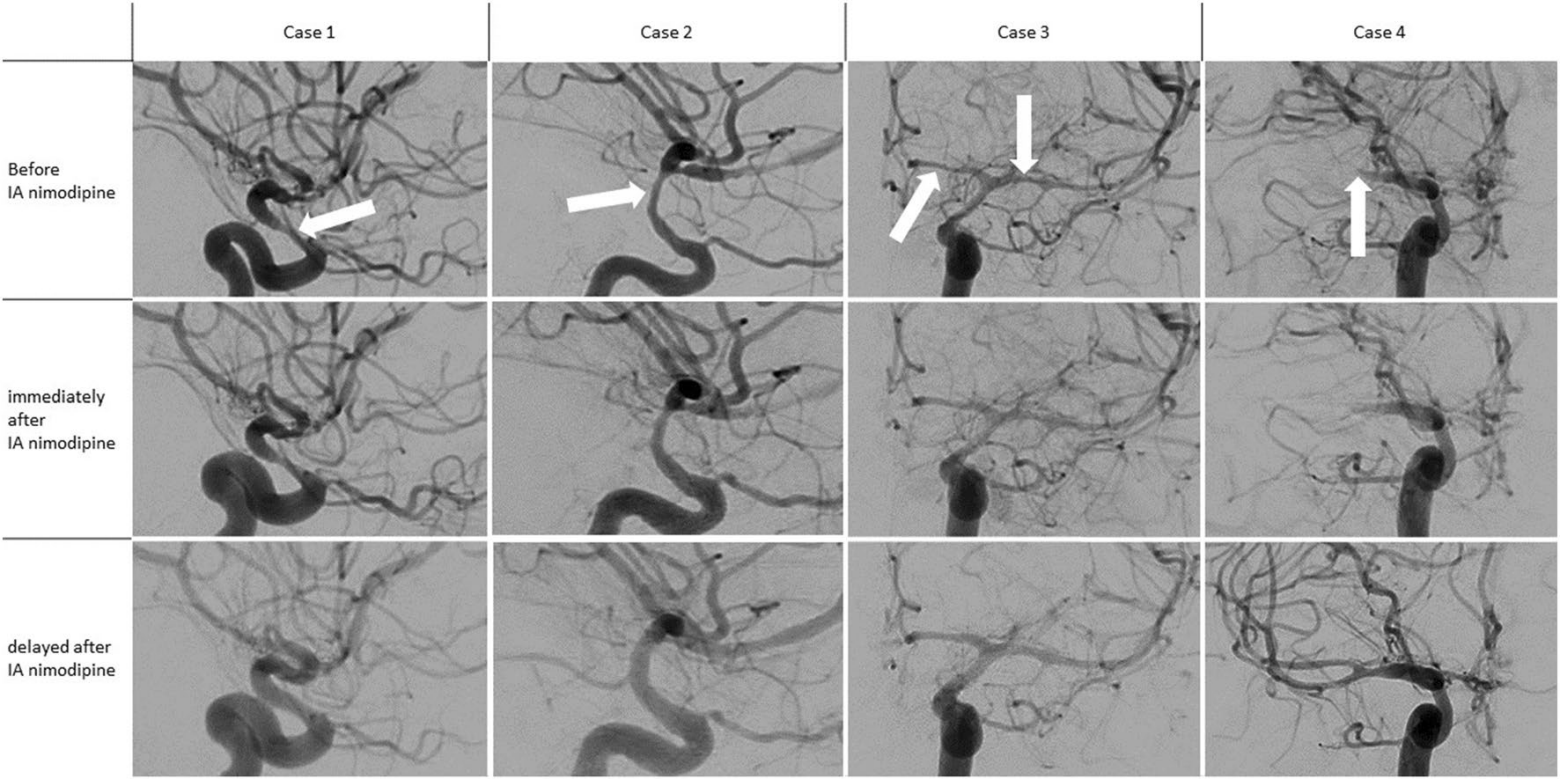
Four cases demonstrating the delayed vasodilatory effect of intra-arterial nimodipine. All cases show a progressive dilation of all vessels. This effect is most pronounced at the site of localized vasospasm (white arrows). While in cases 1, 2 and 3 intra-arterial Nimodipine was infused into other vessels between the immediate control run and the delayed run, in case 4 no additional nimodipine was infused. The occlusive vasospasm of the M1-Segment in case 4 remained unchanged at the time of the immediate control run but was shown to be regressive in the delayed control.

The difference in non-spastic vessel segments was less pronounced with an increase in diameter by 23.1% (± 13.5%) in the delayed controls vs. 13.3% (± 10.7%) in the immediate controls compared to the initial diameter (n = 32; p < 0.0001) (Fig. 3). The angiographic response was rated as insufficient on the immediate controls in 8/32 cases but was rated as sufficient in all delayed controls. A selection of image examples is demonstrated in Fig. 4 as well as in Supplement 1.

Progressive dilation in spastic and non-spastic vessel segments between the immediate and delayed control was independent of the dose of nimodipine administered after the immediate control (spastic vessel segments: rs = 0.3001 (CI − 0.06497–0.5944) (p = 0.0951), non-spastic vessel segments: rs = − -0.1811 (CI − 0.5064–0.1893) (p = 0.3212).

## Discussion

We evaluated the delayed effect of intra-arterial nimodipine as a salvage therapy for cerebral vasospasm after subarachnoid hemorrhage. We found a progressive vasodilation from the immediate to the delayed control in all cases, which was more pronounced in the spastic vessel segments. There was no correlation between vasodilation with the dose of nimodipine administered after the immediate control. The observation that in four cases no nimodipine was administered between the immediate and delayed controls (yet with a progressive vessel dilation in the delayed control) suggests that the observed delayed effect is not due to an increased systemic dose of nimodipine, but that the maximal effect of previously administered nimodipine occurs after a certain delay. Nimodipine is a highly lipid-soluble agent which readily crosses the blood–brain barrier can have a prolonged effect beyond the discontinuation of the intra-arterial infusion^14,15^. This is supported by a study by Lim et al. that demonstrated maximal vasodilation one hour after nimodipine infusion in an animal model, which even persisted for 2 h after intra-arterial infusion^15^. In human studies, the effect of nimodipine was shown to last up to 24 h with improvement of cerebral perfusion in CT perfusion imaging^4^.

Previous studies that used immediate angiographic controls showed a high rate of only poor or moderate changes in vessel diameters after intra-arterial Nimodipine infusion. Hänggi et al. found major angiographic changes only in 8/22 cases (36% of cases)^4^ and angiographic response was judged poor in 57% of cases in a study by Biondi et al.^3^. Nevertheless, the majority (76%) of patients improved clinically after intra-arterial treatment in the same study^3^. The reason for these discrepancies can be explained by our findings suggesting that digital subtraction angiography immediately after administration underestimates the therapeutic effect. A pronounced example of this delayed effect in our cohort was one case with complete occlusion of a vessel at baseline and in the immediate control, which was reopened in the delayed control without further intervention. This should be taken into account when considering further escalation of treatment, for example with a balloon or a retrievable stent, as these alternatives might also be associated with adverse events. New devices and techniques are currently tested for their use in patients suffering from subarachnoid hemorrhage-related cerebral vasospasm and might soon be marketed^16^. Some novel treatment techniques might be judged prematurely as being effective on the false assumption that intra-arterial nimodipine treatment had failed^17^.

Our study has several limitations. First, it was not possible to determine the specific time to the maximum effect of i. a. nimodipine. Taking into account radiation exposure and personnel requirements as well as patient safety, all conventional methods of assessing vasospasm reach their limits in terms of continuous temporal resolution. Continuous monitoring of the patient by means of digital subtraction angiography or CT perfusion is therefore not possible, while transcranial doppler has only a limited sensitivity and negative predictive value respectively^18,19^. The determination of the time to the maximum effect of i. a. nimodipine as well as the temporal course during the decline in effect remain topics for future research projects.

Second, it was not possible to establish a reasonable control group. Performing a DSA on a patient with vasospasms but withholding any i. a.-treatment seems not ethically justifiable. A comparison of nimodipine vs. angioplasty was considered but rejected due to the reasons that (1) angioplasty can only be considered as a stand-alone therapy in the context of singular, proximal vasospasms, and (2) the patient's neurological status is not influenced by the vasospasms alone, but also by peripheral, molecular processes, which may not be reached by simple mechanical angioplasty.

Third, the present study focuses only on intra-arterial drug therapy with nimodipine, without comparing it to verapamil or nicardipine. Although these other drugs are used more frequently in other centers, it should be noted that in previous studies, nimodipine was proven to be one of the most effective treatments (especially in comparison to nicardipine)^20^, with a longer lasting effect in comparison to verapamil in animal studies^15^, which makes it the drug of choice in our center.

Fourth, a possible influence of administered volatile anesthetics and noradrenaline on vasodynamics cannot be distinguished from the influence of nimodipine. Since norepinephrine is thought to have a rather constricting effect on the cerebral vasculature, a relevant influence on the described results (with progressive vasodilation) seems possible, but less relevant than the influence of volatile anesthetics. As for volatile anesthetics, several studies have shown that they attenuate both angiographic vasospasm and clinical deterioration after subarachnoid hemorrhage (in the setting of delayed cerebral ischemia), so that an influence on the results of our study seems likely. To distinguish the possible influence of volatile anesthetics from the influence of nimodipine, further prospective studies are needed, e.g. comparing vasodilation during general anesthesia with volatile anesthetics vs. general anesthesia with propofol.

Some further limitations are the retrospective and single-center-design of the study, as well as the small sample size.

The delayed controls were established in our department with the intention to escalate endovascular treatment if nimodipine infusion was deemed insufficient. Judging by the immediate controls 25.0% of cases showed an insufficient response to the treatment that would have triggered an angioplasty with a balloon or a retrievable stent. Yet not a single case in our cohort was treated or would have retrospectively been treated with angioplasty after reviewing the delayed control.

Intra-arterially administered nimodipine seems to exert a delayed vasodilatory effect on spastic vessel segments more than on non-spastic vessel segments. This effect should be considered before judging vasodilatory therapy to be unsuccessful and expand treatment towards angioplasty.

### Supplementary Information

Supplementary Information.

## Data Availability

The datasets generated during and/or analysed during the current study are not publicly available due to ensure individual privacymbut are available from the corresponding author on reasonable request.

## Abbreviations

ACOM: Anterior communicating artery
ICA: Internal carotid artery
MCA: Medial cerebral artery
mRS: Modified Ranking scale
PCA: Posterior cerebral artery
PICA: Posterior inferior cerebellar artery
