# Supplementary material for: Immediate angiographic control after intra-arterial nimodipine administration underestimates the vasodilatory effect
Source: Sci Rep. 2024 Mar 14;14:6154. doi: 10.1038/s41598-024-56807-7 (PMC10940303; doi:10.1038/s41598-024-56807-7)
Supplement: Supplementary file 1 — Supplementary Information. [file 41598_2024_56807_MOESM1_ESM.pdf]

Supplement:

Exemplary cases of vasospasmolysis of two patients in the course of repeated interventions

before IA nimodipine | immediately after IA nimodipine | delayed after IA nimodipine

patient 1 case 1

patient 1 case 2

patient 1 case 3

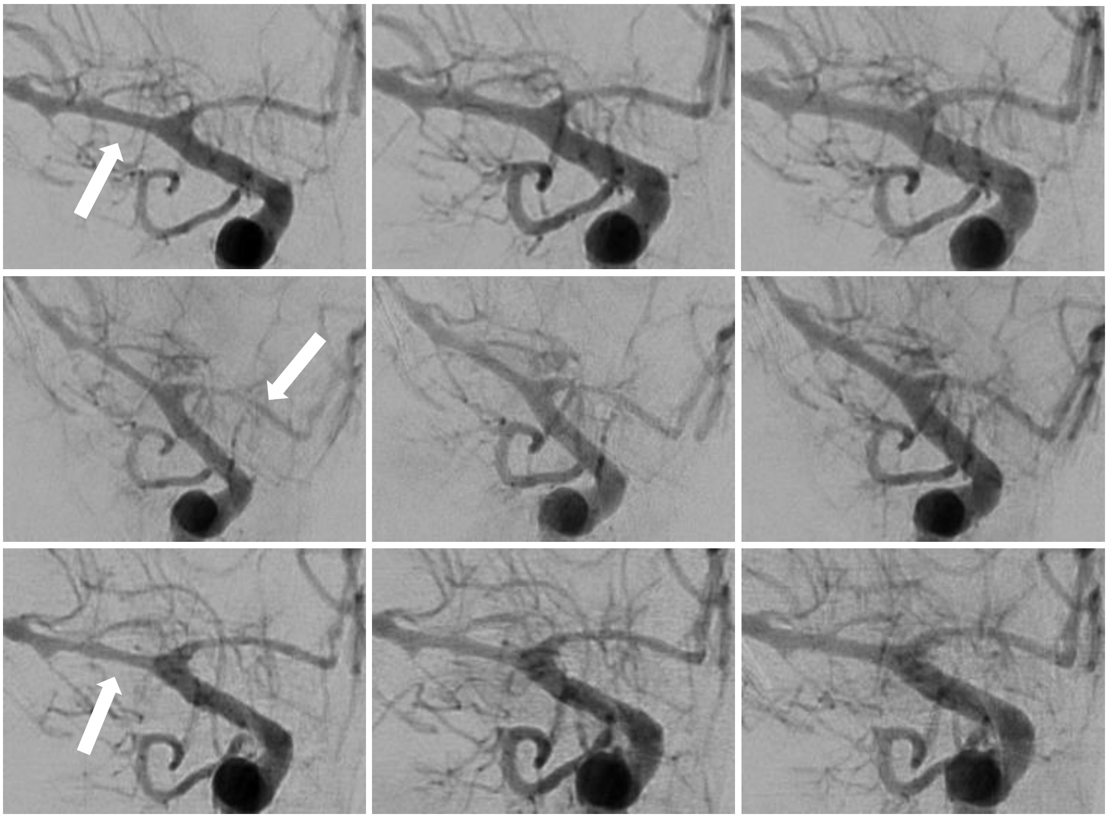

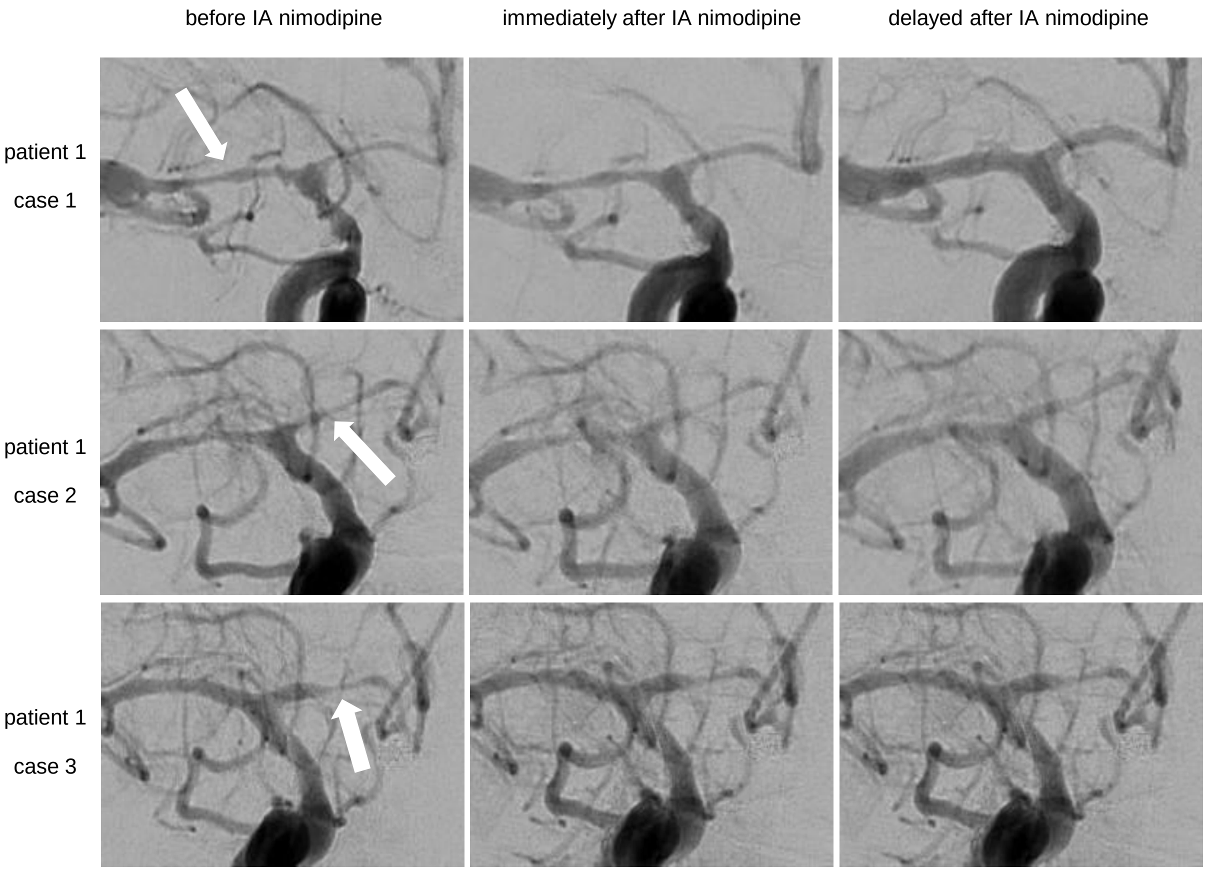
before IA nimodipine
immediately after IA nimodipine
delayed after IA nimodipine
patient 1
case 1
patient 1
case 2
patient 1
case 3
